# Integrated weighted gene co-expression network analysis uncovers STAT1(signal transducer and activator of transcription 1) and IFI44L (interferon-induced protein 44-like) as key genes in pulmonary arterial hypertension

**DOI:** 10.1080/21655979.2021.1972200

**Published:** 2021-09-13

**Authors:** Han Yang, Yang Lu, Hongmin Yang, Yaoxi Zhu, Yaohan Tang, Lixia Li, Changhu Liu, Jing Yuan

**Affiliations:** Department of Cardiology, Union Hospital, Tongji Medical College, Huazhong University of Science and Technology, Wuhan, China

**Keywords:** Pulmonary arterial hypertension, STAT1, IFI44L, gene expression, WGCNA

## Abstract

Despite the multiple diagnostic and therapeutic strategies implemented in clinical practice, the mortality rate of patients with pulmonary arterial hypertension (PAH) remains high. Understanding the mechanisms and key genes involved could provide insight into the drivers of the pathogenesis of PAH. In this research, we aimed to examine the mechanisms underlying PAH and identify key genes with potential usefulness as clinical biomarkers of PAH and thereby establish therapeutic targets for PAH. The datasets GSE117261, GSE113439, and GSE53408 were downloaded from the Gene Expression Omnibus (GEOs) database. We used weighted gene coexpression network analysis (WGCNA) to identify networks and the most relevant modules in PAH. Functional enrichment analysis was performed for the selected clinically relevant modules. The least absolute shrinkage and selection operator (LASSO) was applied to identify key genes in lung samples from patients with PAH. The genes were validated in a monocrotaline-induced PAH rat model. Three clinically relevant modules were identified through average linkage hierarchical clustering. The genes in the clinically relevant modules were related to endothelial cell differentiation, inflammation, and autoimmunity. Seven genes were screened as key genes significantly associated with PAH. Interferon-induced protein 44-like (IFI44L) and signal transducer and activator of transcription 1 (STAT1) were expressed at higher levels in the lung tissues of the PAH rat model than in those of the controls. Our findings reveal the novel pathological mechanisms underlying PAH and indicate that STAT1 and IFI44L may represent potential therapeutic targets in PAH.

## Introduction

Pulmonary arterial hypertension (PAH) is an incurable disease characterized by endothelial dysfunction and dysregulated pulmonary vascular smooth muscle cells and fibroblasts proliferation [1]. The recent progress in the research on the pathological mechanisms underlying PAH has led to the development of targeted therapies. Strategies targeting crucial pathways, including nitric oxide, prostacyclin, RhoA/Rho kinase pathway modulators, and endothelin-1, have been used in patients with PAH [2]. Although the current PAH-targeted therapies can improve the quality of life and reduce the hospital readmission rate, the 5-year mortality rate of patients with PAH remains approximately 50% [3]. Therefore, a deeper understanding of the molecular mechanisms underlying PAH may enable the development of novel drugs to improve the survival rate of patients.

Weighted gene co-expression network analysis (WGCNA) is a bioinformatics method that facilitates network-based gene screening to identify novel biomarkers or therapeutic targets [4]. In recent years, studies have aimed to examine the molecular mechanisms and screen hub genes related to PAH on the basis of differentially expressed genes (DEGs) [5,6]. However, the use of WGCNA to identify genes involved in PAH remains limited. Therefore, we used the mRNA expression profiles of patients with PAH to perform a WGCNA for the identification of networks significantly associated with PAH. Understanding the mechanisms and key genes involved could provide insight into the drivers of the pathogenesis of PAH. In this research, we aimed to examine the underlying pathological mechanisms of PAH to identify targets for early diagnosis and treatment of PAH. In addition, we investigated the relationship between hub genes and infiltrating immune cells to better understand the immunological mechanisms that contribute to the development of PAH.

## Methods

### Data resources

The mRNA expression profiles of patients with PAH were obtained from the Gene Expression Omnibus (GEOs) (https://www.ncbi.nlm.nih.gov/geoprofiles/) [7]. Datasets GSE117261 [8] (58 patients with PAH and 25 controls), GSE113439 [9] (15 patients with PAH and 11 controls), and GSE53408 [10] (12 patients with PAH and 11 controls) were analyzed. Moreover, the GSE48149 [11] dataset was used as the validation set.

### Construction of the gene coexpression network

A coexpression network of 4000 genes with the highest median absolute deviation was constructed using WGCNA [12,13]. Sample outliers were identified by clustering and removed. A power of *β* = 9 (scale-free *R*^2^ = 0.96) was selected to ensure a scale-free topology. Hierarchical clustering was performed by constructing a topological overlap matrix, and genes with coexpression relationships were grouped through gene network connectivity. The minimum cutoff size was set to 30. Highly similar gene modules were further analyzed. The relationship between clinical features and gene modules was evaluated with the Pearson correlation analysis to identify biologically meaningful modules.

### Gene ontology and pathway enrichment analyses

Metascape (https://metascape.org/gp/index.html) [14] was used to assess the biological functions of genes upregulated or downregulated in patients with PAH. Gene ontology (GO) and Kyoto Encyclopedia of Genes and Genomes (KEGG) pathway enrichment analyses were performed. A *P* value < 0.01 was considered to indicate statistical significance.

### Protein-protein interaction network and hub protein screening

STRING (http://string-db.org) [15,16] was used to construct the protein-protein interaction (PPI) network of modules with a confidence score > 0.40. Hub genes were identified using the Cytoscape software V3.5.1 (http://cytoscape.org) [17]. Plug-in CytoHubba was used to detect the top 30 hub genes in each clinically significant module identified by WGCNA.

### Screening of key genes

RNA expression data were processed using the ‘SVA’ package [18] and the ‘ComBat’ algorithm in R.4.0.3. Then, we used the ‘limma’ package [19] to evaluate the DEG between the PAH and control groups. Genes with adjusted P values < 0.05 were considered as DEG. The top 30 hub genes in each clinically significant module were added together and crossed with the DEGs. We then used the least absolute shrinkage and selection operator (LASSO) logistic regression to identify the key genes [20]. In addition, a receiver-operating characteristic (ROC) curve analysis was performed to evaluate the stability and sensitivity of the LASSO model in identifying PAH by using the pROC package in R software version 3.6.0 [21].

### Evaluation of immune cell infiltration

We used the gene expression matrix of xCell (https://xcell.ucsf.edu) to obtain the immune cell infiltration matrix [22]. The Wilcoxon method was used to analyze the variance, and the cutoff P value for the cell analyses was <0.05. Only immune cells were included in the analysis. The ‘ggplot2’ package was used to create violin diagrams to illustrate the differences in immune cell infiltration.

### Construction of the monocrotaline-induced PAH rat model

We randomly divided male Sprague-Dawley (SD) rats (weight, 180–220 g; age: 4–6 weeks; SPF class) into two groups, the control (n = 6) and PAH groups (n = 6). The rats in the PAH group were intraperitoneally injected with monocrotaline (MCT; 60 mg/kg) on day 0. The control rats were intraperitoneally injected with the same volume of saline on day 0. The mean pulmonary arterial pressure of the rats was measured as the mean right ventricular systolic pressure (mRVSP) by right heart catheterization at 4 weeks after intraperitoneal injection. All the rats were sacrificed 4 weeks after MCT administration. All the animal experiments were performed in accordance with the National Institutes of Health Guide for the Care and Use of Laboratory Animals.

### Real-time polymerase chain reaction (PCR)

Total RNA was extracted from frozen lung sections using the TRIzol reagent (Invitrogen) and reverse-transcribed into cDNA with a PrimeScript RT reagent kit (TaKaRa Biotechnology). The relative gene expression was calculated using the 2^−ΔΔCt^ method. GAPDH was used as the reference housekeeping gene. The sequences of the primers were as follows: HIST1H1C (F: 5'-GAAGCCCAAGAAGGCTACGG-3'; R: 5'-GCTTTGGGCTTTACCGCTCT-3'), IFI44L (F: 5'-GCTGTGTGATTCAATGGGGC-3'; R: 5'-GGAGAGGCAGCGTAAGTGAA-3'), STAT1 (F: 5'-GAACGTGCTCTGCTCAAGGA-3'; R: 5'-AACAGCATGGAAGTCGGGTT-3'), TAF4B (F: 5'-CCTGCGGTGACAAGTACAGT-3'; R: 5'-TTGCGGTCCACCATTAGCAG-3'), PRKAR2B (F: 5'-TGGAAATCGCTCGGTGTCTC-3'; R: 5'-TCATGCAGTGGGCTCAACAA-3'), CEP57 (F: 5'-CATCGGGGACTTGGATTCGG-3'; R: 5'-TCAGCTAGGACGCTCGAAAA-3'), MLLT3 (F: 5'-ATGTCCGCCATCTACCCTCA-3'; R: 5'-CCATCCAGTCGTGGGTGAAG-3'), GAPDH (F: 5'-GAAGGTCGGTGTGAACGGAT-3'; R: 5'-CCCATTTGATGTTAGCGGGAT-3'). Real-time PCR was conducted using the SYBR Premix Ex Taq kit (TaKaRa Biotechnology) and an ABI PRISM 7900 Sequence Detector System (Applied Biosystems).

## Results

In this research, we aimed to explore the mechanisms underlying PAH and identify key genes that may be employed as potential clinical biomarkers and thereby provide therapeutic targets for PAH. Three clinically relevant modules were identified through the WGCNA. The GO and KEGG enrichment analyses revealed that the genes in the clinically relevant modules were related to endothelial cell differentiation, inflammation, and autoimmunity. The LASSO was applied to identify key genes, and seven genes were screened as key genes significantly associated with PAH. Besides, IFI44L and STAT1 were expressed at higher levels in the lung tissues of the PAH rat model than in those of the controls. Our findings reveal the novel pathological mechanisms underlying PAH and indicate that STAT1 and IFI44L may represent potential therapeutic targets in PAH. The research flow chart is represented in the .

### Construction of gene coexpression modules

**Figure 1.**
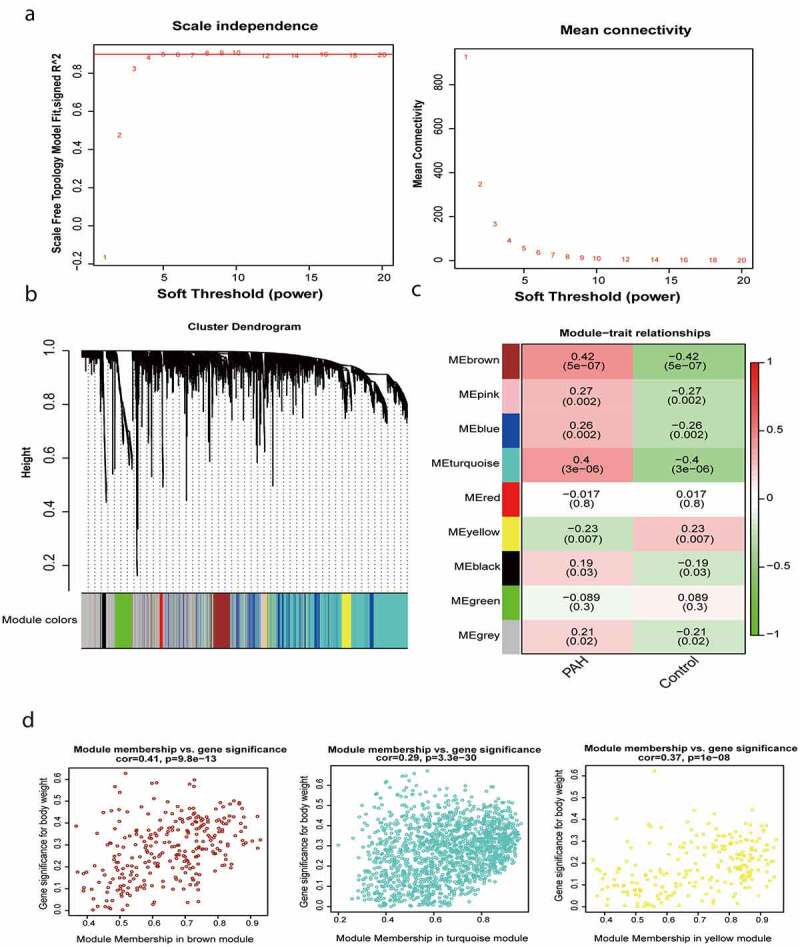
Construction of gene co-expression modules. (a) Analysis of network topology for various soft-thresholding powers. (b) Gene clustering tree (dendrogram) obtained by hierarchical clustering of adjacency-based dissimilarity. (c) Module-feature associations. Each row corresponds to a module eigengene, and each column corresponds to a clinical feature. Red represents a positive correlation, and green represents a negative correlation. The darker the corresponding color, the stronger the correlation coefficient. (d) Scatter plot of eigengene modules

The gene expression profiles of the human lung tissues from 45 healthy individuals and 85 patients with severe PAH were evaluated. Expression data were normalized, and 4000 genes with the most significant changes (highest median absolute deviations) were used for WGCNA analysis. After removing outliers (Supplementary Figure 2), a soft-threshold filtering approach (soft-threshold *β* = 9) was used to construct a scale-free network using the WGCNA package (Figure 1a). In accordance with the weighted correlation, we analyzed the hierarchical clustering and segmented the clustering results in accordance with the set criteria. A total of nine gene modules were obtained, which are represented by the branches and different colors of the clustering tree shown in Figure 1b.

### Identification of key modules

We calculated the correlation between the gene modules and the phenotype and identified three module eigengenes (shown in brown, turquoise, and yellow) strongly associated with PAH (Figure 1c). The module membership and gene significance values were as follows: brown module, cor = 0.41 and P = 9.8e-13; turquoise module, cor = 0.29 and P = 3.3e-30; and yellow module, cor = 0.37 and P = 1e-08 (Figure 1d). It should be noted that in WGCNA, the gray module always represents background genes (noise genes) outside the modules, with no distinct module assignment [8]. Therefore, a certain percentage of gray module is normal and acceptable.

### GO and pathway enrichment analyses

**Figure 2.**
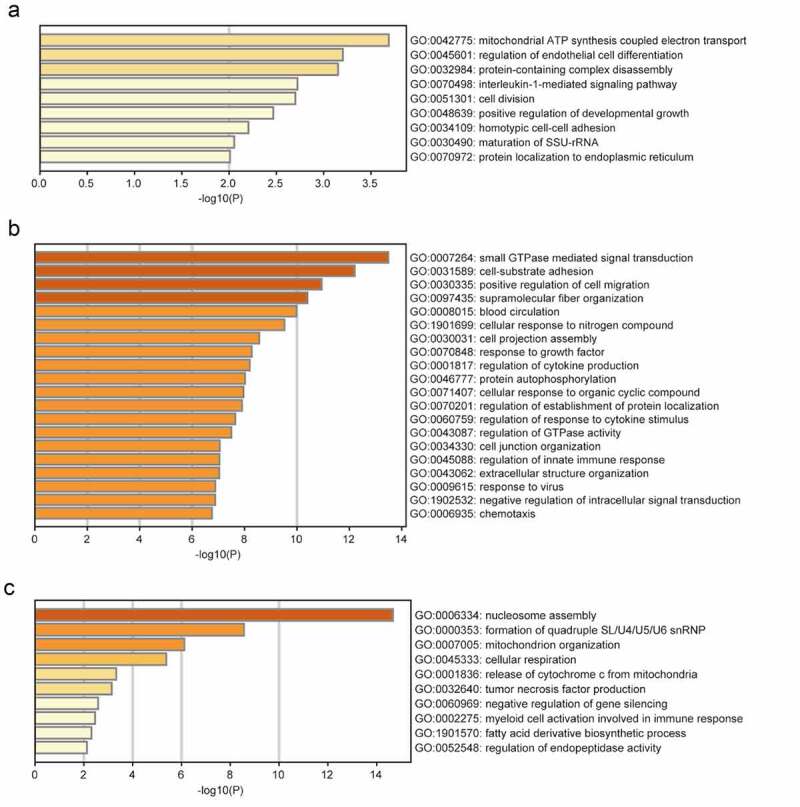
GO functional analysis of the profiles. GO biological processes of genes in brown module (a), turquoise module (b), yellow module (c)

**Figure 3.**
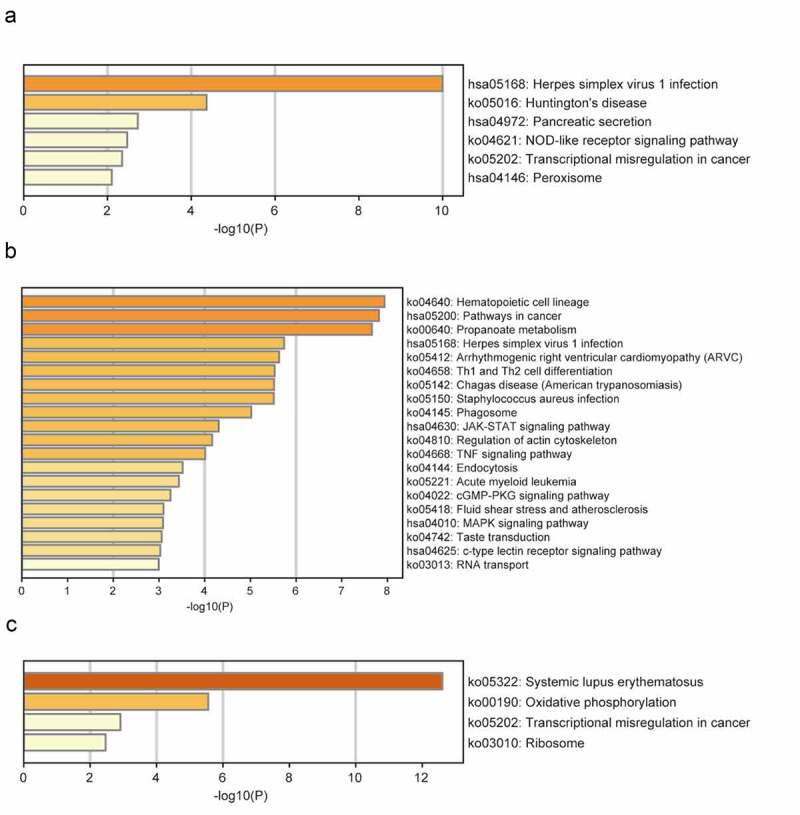
KEGG pathway analysis of the profiles. KEGG enrichment analysis of genes in brown module (a), turquoise module (b), yellow module (c)

The brown module was significantly enriched in GO biological processes associated with mitochondrial adenosine triphosphate (ATP) synthesis-coupled electron transport, regulation of endothelial cell differentiation, protein-containing complex disassembly, interleukin-1-mediated signaling, and positive regulation of developmental growth (Figure 2a). The enriched KEGG pathways included herpes simplex virus 1 (HSV1) infection and NOD-like receptor signaling (Figure 3a). The turquoise module was significantly enriched in GO biological processes related to small GTPase-mediated signal transduction, positive regulation of cell migration, response to growth factors, regulation of cytokine production, regulation of response to cytokine stimulus, and regulation of innate immune responses (Figure 2b). The enriched KEGG pathways included HSV1 infection, Th1 and Th2 cell differentiation, Janus kinase (JAK)-STAT signaling, and tumor necrosis factor (TNF) signaling (Figure 3b). The significantly enriched GO biological processes in the yellow module included the nucleosome assembly, cellular respiration, TNF production, negative regulation of gene silencing, and myeloid cell activation involved in immune responses (Figure 2c). The enriched pathways included systemic lupus erythematosus, oxidative phosphorylation, and transcriptional dysregulation in cancer (Figure 3c).

### Identification of hub genes

**Figure 4.**
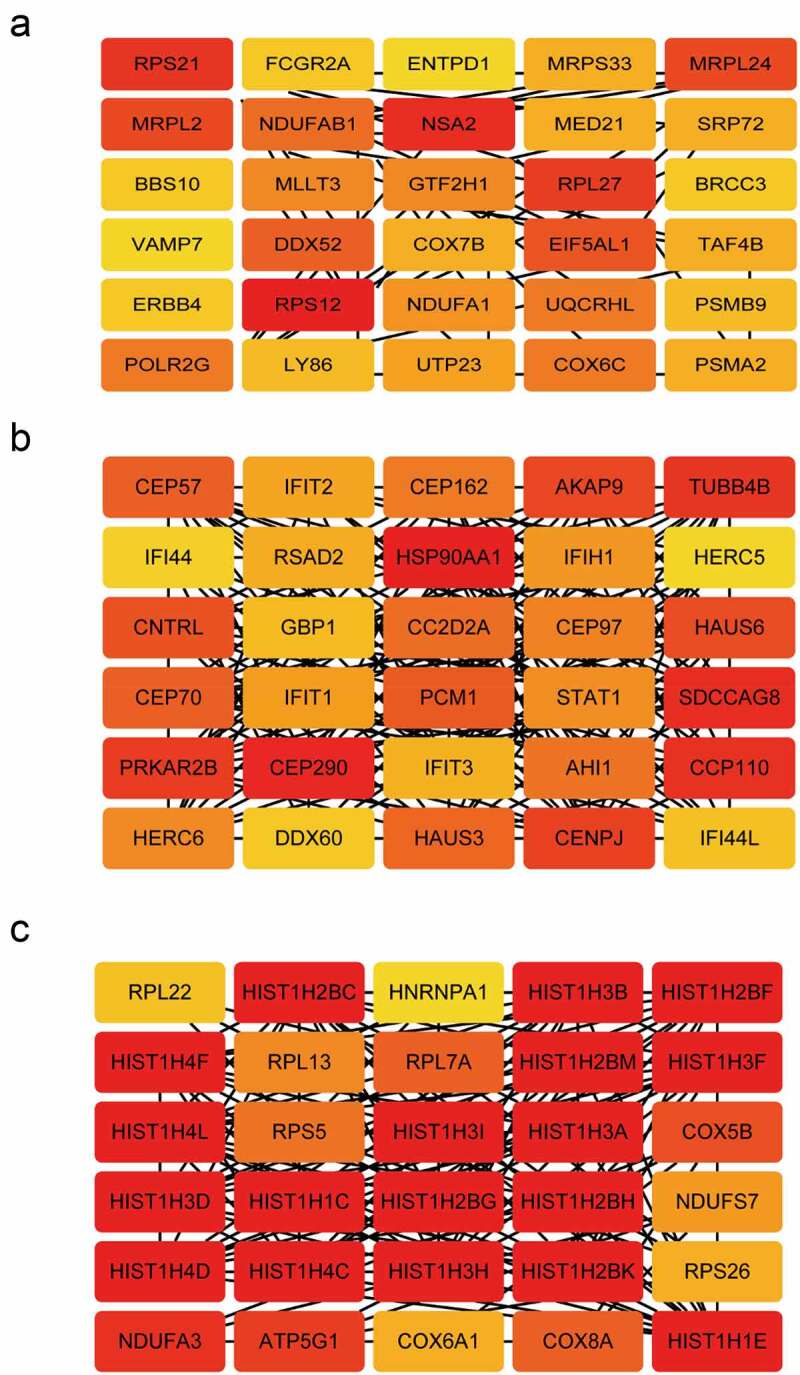
Identification of hub genes. The network of hub genes in the (a) brown module, (b) turquoise module, (c) yellow module. Red represents a positive correlation, The darker the color, the stronger the correlation

From the three modules, the top 30 genes with the highest module membership values in each module were selected as hub genes. We obtained a total of 90 candidate genes (Figure 4).

### Screening and verification of the key genes

**Figure 5.**
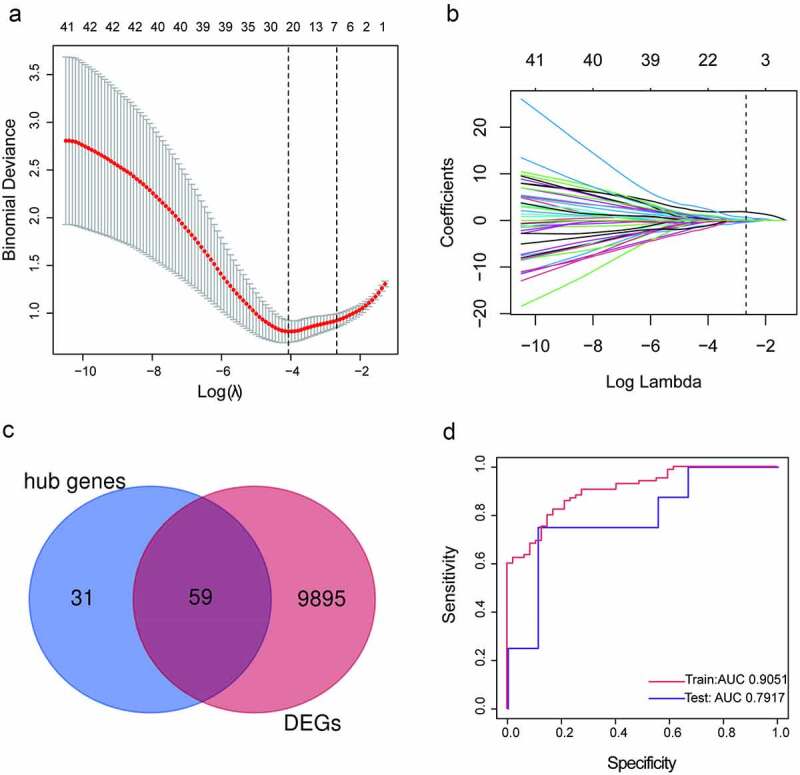
Screening and verification of key genes. (a-b) Least absolute shrinkage and selection operator (LASSO) logistic regression algorithm to screen key genes.(c) Venn diagram. Blue section stands for hub genes, pink section stands for differentially expression genes (DEGs), 59 genes in the middle overlapping section. (d) Receiver operating characteristic (ROC) curves analysis of training set and testing set. AUC, area under the curve

To determine the most relevant genes in the development of PAH, we identified the DEGs between the specimens from the patients with PAH and those from healthy volunteers and yielded 9954 DEGs with adjusted P values < 0.05. Then, we compared the 90 hub genes and 9954 DEGs and obtained 59 common genes (Figure 5c). LASSO logistic regression revealed that seven (*HIST1H1C, IFI44L, STAT1, TAF4B, PRKAR2B, MLLT3*, and *CEP57*) of the 59 genes were key genes in the development of PAH (Figure 5a, b), with coefficients of −0.22915, 0.055855, 0.083195, 0.204453, 0.108119, 0.32228, and 0.099729 for *HIST1H1C* (Histone H1c), *IFI44L* (interferon-induced protein 44-like), *STAT1(Signal transducer and activator of transcription 1), TAF4B* (TATA-box binding protein associated factor 4b), *PRKAR2B* (cAMP-dependent protein kinase type II-beta regulatory subunit), *MLLT3* (mixed-lineage leukemia translocated to chromosome 3 protein), and *CEP57* (centrosomal protein 57), respectively. A ROC curve analysis of the LASSO regression model was conducted to predict PAH in the training set, and the area under the curve (AUC) was 0.9051. To further test the diagnostic efficacy, we validated it with the GSE48149 dataset as the testing set, and the AUC was 0.7917, which suggests that the genes in the model might have high diagnostic values (Figure 5d). The diagnostic efficacy of the seven key genes are represented in Supplementary Figure 3.

### Validation of the key genes in the rat PAH model

**Figure 6.**
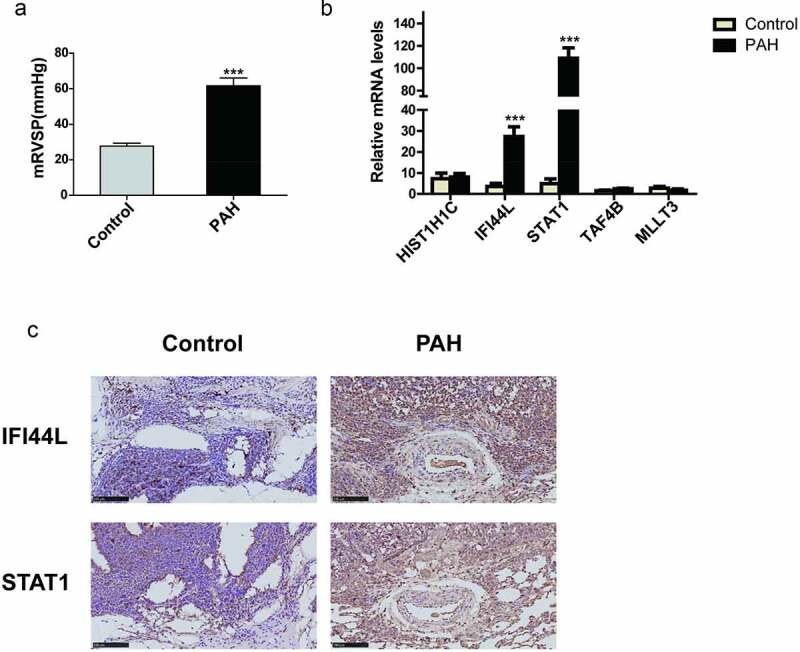
Validation of the key genes in a rat PAH model (a) Mean right ventricular systolic pressure (mRVSP) in control and MCT-induced PAH rats. (b) The levels of *HIST1H1C*, *IFI44L*, *STAT1*, *TAF4B*, and *MLLT3* mRNA expression in lung tissues from control and MCT-induced PAH rats; (c) The expression of IFI44L and STAT1 in the lung tissue of the control group and PAH group. Representative immunostaining images of lung sections show the expression of IFI44L and STAT1 increased in the lung tissues of MCT-induced PAH rats. Scales bars, 50 μm for high-resolution images (original magnificationⅹ400). N = 6 for the control group and PAH group. Compared with the control group, ***P < 0.001. Data presented in A and B are mean ± SEM of three independent experiments. PAH, pulmonary arterial hypertension

The mean right ventricular systolic pressure (mRVSP) of the rats in the PAH group was significantly higher than that of the rats in the control group (P < 0.001; Figure 6a). To confirm the clinical significance of the seven genes, we evaluated their mRNA levels in the lung tissues from the MCT-PAH or control rats. *IFI44L* and *STAT1* were expressed at higher levels in the PAH lung tissues than in the control lung tissues (P < 0.001), whereas *HIST1H1C, TAF4B*, and *MLLT3* showed no significant differences in expression level between the two groups (Figure 6b). The mRNAs of *PRKAR2B* and *CEP57* could not be detected. We also performed immunohistochemical staining of rat lung tissues for STAT1 and IFI44L. The STAT1 and IFI44L expression levels were profoundly higher in the lung tissues of the PAH rats than in those of the control rats (Figure 6c).

### Immune cell infiltration

**Figure 7.**
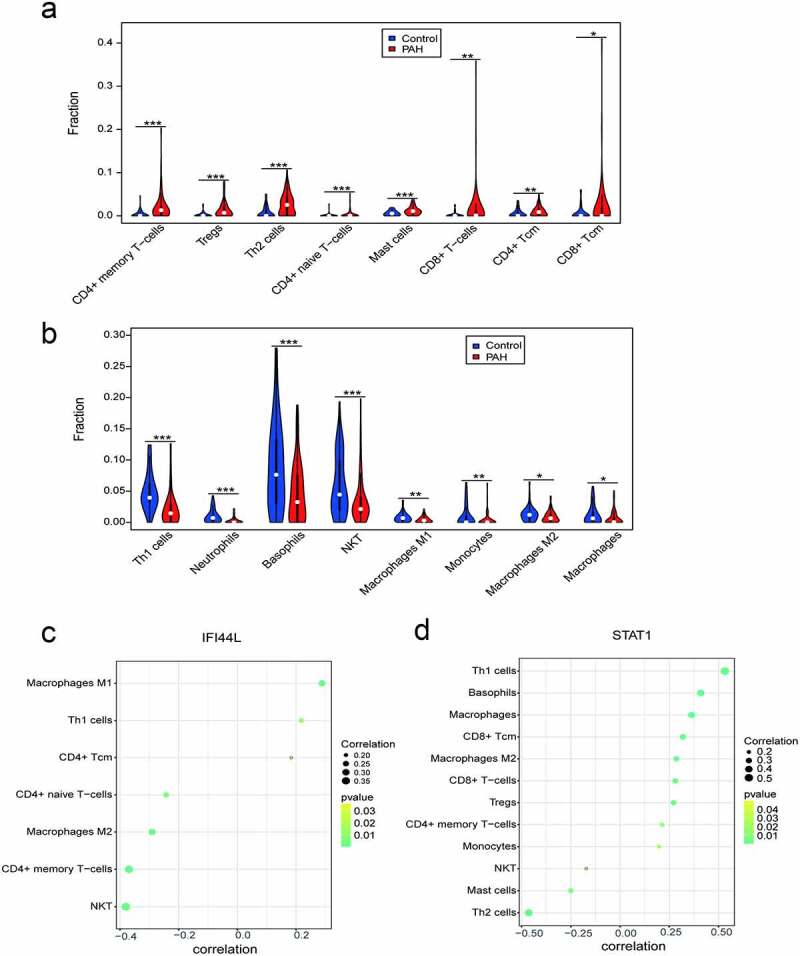
Different immune cell infiltration between PAH and control group. (a) Violin diagram of the proportion of immune cells which infiltrated more in PAH group than in control group; (b) Violin diagram of the proportion of immune cells which infiltrated less in PAH group than in control group. ***P < 0.001, **P < 0.01, *P < 0.05. Correlation between IFI44L, STAT1, and infiltrating immune cells. (c) Correlation between IFI44L and infiltrating immune cells. (d) Correlation between STAT1 and infiltrating immune cells. PAH, pulmonary arterial hypertension

The levels of CD4^+^ memory T cells, regulator T cells (Tregs), Th2 cells, CD4^+^-naive T cells, mast cells, CD8^+^ T cells, CD4^+^ central memory T (Tcm) cells, and CD8^+^ Tcm cells were higher in the PAH tissues than in the control samples (Figure 7a). By contrast, the levels of Th1 cells, neutrophils, basophils, NKT cells, M1 macrophages, monocytes, M2 macrophages, and macrophages were lower in the PAH tissues than in the control samples (Figure 7b).

### Relationships between IFI44L, STAT1, and immune cell infiltration

The correlation analysis revealed that the IFI44L levels were significantly associated with the levels of M1 macrophages, Th1 cells, and CD4^+^ Tcm cells. By contrast, the IFI44L levels negatively correlated with the levels of CD4^+^-naive T cells, M2 macrophages, CD4^+^ memory T cells, and NK T cells (Figure 7c). STAT1 levels were positively associated with Th1 cells, basophils, and macrophages, but negatively correlated with the levels of NKT, mast cells, and Th2 cells (Figure 7d).

## Discussion

In this study, we performed a WGCNA and identified nine gene modules associated with PAH. The enrichment analyses revealed that the most enriched GO terms of the biological processes in the brown module were associated with cell metabolism and endothelial cell differentiation and inflammation. Studies have shown that mitochondrial metabolic dysfunction in pulmonary arterial endothelial cells, pulmonary artery smooth muscle cells (PASMCs), and ventricular cardiomyocytes was linked to PAH progression [23–25]. The identified GO category ‘regulation of endothelial cell differentiation’ fits well with the concept emphasizing that endothelial cell dysfunction is one of the most important factors predisposing to vascular remodeling and pulmonary vascular occlusion in PAH. Moreover, IL-1β stimulation in PASMCs has been shown to promote inflammation, proliferation, and migration [26]. The significantly enriched functions in the turquoise module were associated with small GTPase-mediated signal transduction. The most well-studied small GTPase in PAH is Rho-kinase belonging to the Ras superfamily. Activation of Rho-kinase in PAH induces calcium sensitization, which contributes to the contractile, cell proliferation, and anti-apoptotic phenotype of PASMCs [27,28]. The enriched KEGG pathways in the turquoise module included the signaling pathways involved in inflammation, immunity, cell migration, and proliferation. These pathways included the JAK-STAT signaling pathway, TNF signaling pathway, and mitogen-activated protein kinase (MAPK) signaling pathway. These findings suggest that these signaling pathways play a critical role in the inflammatory responses in PAH. Recent studies have shown that *in vitro* inhibition of Jak1 and Jak2 with ruxolitinib reduced the proliferation and migration of PASMCs in patients with PAH in a dose-dependent manner [29]. These signaling pathways, which are associated with PAH, could be targeted therapeutically for the prevention and treatment of PAH. HSV1 signaling was significantly enriched among the genes in the brown and turquoise modules. The genes in the yellow module were related to nucleosome assembly, mitochondrial organization, and cellular respiratory, all of which may have a negative correlation with PAH.

Moreover, seven potential hub genes associated with PAH were screened, namely *HIST1H1C, IFI44L, STAT1, TAF4B, PRKAR2B, MLLT3*, and *CEP57*. Research on the role of these genes in PAH is limited, and future investigations are required to elucidate their clinical relevance in PAH. We found that among these genes, IFI44L and STAT1 were the most prominent DEGs between the lung tissues of the PAH rats and those of the control rats. IFI44L belonging to the IFI44 family is a type I interferon-stimulated gene (ISG) [30]. IFI44L has been considered to play a role in inflammation, autoimmune disorders, and cancer [31]. Recent evidence suggests IFI44L as a novel tumor suppressor regulating cancer stemness and metastasis in hepatocellular carcinoma by modulating Met/Src signaling [32]. Using IFI44L expression levels, Weeding et al. distinguished individuals with systemic lupus erythematosus (SLE) from healthy controls. They also found that IFI44L hypomethylation was a highly sensitive and specific marker of SLE [33,34]. Recently, Busse et al. and Marta et al. demonstrated that IFI44L negatively regulated innate immune responses induced by viral infections and could be a promising therapeutic target for diseases associated with excessive IFN-driven proinflammatory responses [30,35].

STAT1 is a nuclear transcription factor that plays an important role in the cell cycle, cell apoptosis, and immune responses [36]. In this study, we found that the JAK-STAT signaling pathway was significantly enriched in the turquoise gene module. Previous studies showed that STAT3 is one of the main intracellular transcription factors involved in PAH-associated vascular remodeling [37]. Recent studies have also shown that IL-6 initiated JAK-STAT signaling to induce the transcription of proinflammatory and pro-angiogenic genes, thereby promoting PAH development [38]. The IFN-STAT1 pathway can promote inflammation and endothelial dysfunction via several mechanisms, including the stimulation of proinflammatory cytokine expression and the production of reactive oxygen species and nitric oxide [39]. The IFN-STAT1 pathway also promotes the release of monocyte chemoattractant protein −1 (MCP-1) from monocytes; in turn, MCP-1 promotes PAH progression by recruiting leukocytes and activating immune cells at sites of vascular inflammation [39,40]. These findings indicate that IFI44L and STAT1 may represent promising therapeutic targets for PAH.

Altered inflammation and immune responses are increasingly recognized as key pathological drivers of PAH. In this study, we used xCell to comprehensively evaluate the immune infiltration in lung tissues from patients with PAH to obtain a deeper understanding of the inflammatory components associated with the pathogenesis of PAH. We found that the lung tissues from the patients with PAH had increased levels of infiltrating Th2 cells, Tregs, and mast cells and decreased levels of macrophages. PAH is associated with immune dysregulation characterized by increased perivascular infiltration of T cells and mast cells and dysfunction of Th2 cells and Tregs [3]. Pathogenic Th2 cells have been shown to promote pulmonary artery muscularization by secreting IL-13 [41]. Treg cells have been shown to maintain pulmonary vascular homeostasis by attenuating vascular inflammation, thereby preventing vascular injury [42]. In addition, the accumulation of macrophages in pulmonary arterioles has been characterized as a prominent pathological feature of PAH. We found decreased infiltration of macrophages in the lung tissues from the patients with PAH. Resident macrophages in the alveoli and interstitium maintain an anti-inflammatory microenvironment by secreting IL-10, which prevents the activation of Th1 and Th17 inflammatory responses in the lungs [41,43]. Alveolar macrophages that tend to be anti-inflammatory and regenerative are depleted in the setting of PAH. The decreased infiltration of macrophage cells may be attributed to the loss of interstitial resident macrophages in our samples.

We also analyzed the relationship between *IFI44L, STAT1*, and immune cells and found that *IFI44L* expression levels positively correlated with the levels of macrophages M1 and Th1 cells. Similarly, STAT1 expression levels were positively associated with the levels of macrophages and Th1 cells and negatively associated with mast cells. Therefore, IFI44L and STAT1 may be involved in signaling pathways related to the recruitment of inflammatory cells. These hypotheses require further research to better understand the complex interactions between genes and immune cells.

## Conclusion

In conclusion, we identified novel signaling pathways, immune profiles, and seven key genes associated with the development of PAH. The experimental validation using lung tissue samples from a PAH rat model revealed that IFI44L and STAT1 are the most relevant genes and may be used as novel biomarkers of PAH. However, some important limitations remain in our study. Information on the diagnostic, inclusion criteria, and the treatments for the PAH patients registered in the database was not available. Another important limitation is the lack of validation of the discovered key genes in lung tissue samples from PAH patients.

## Supplementary Material

Supplemental Material
