# Supplementary material for: Integrated weighted gene co-expression network analysis uncovers STAT1(signal transducer and activator of transcription 1) and IFI44L (interferon-induced protein 44-like) as key genes in pulmonary arterial hypertension
Source: Bioengineered. 2021 Sep 13;12(1):6021–34. doi: 10.1080/21655979.2021.1972200 (PMC8806536; doi:10.1080/21655979.2021.1972200)
Supplement: Supplemental Material [file KBIE_A_1972200_SM8298.docx]

**Figure and legends**


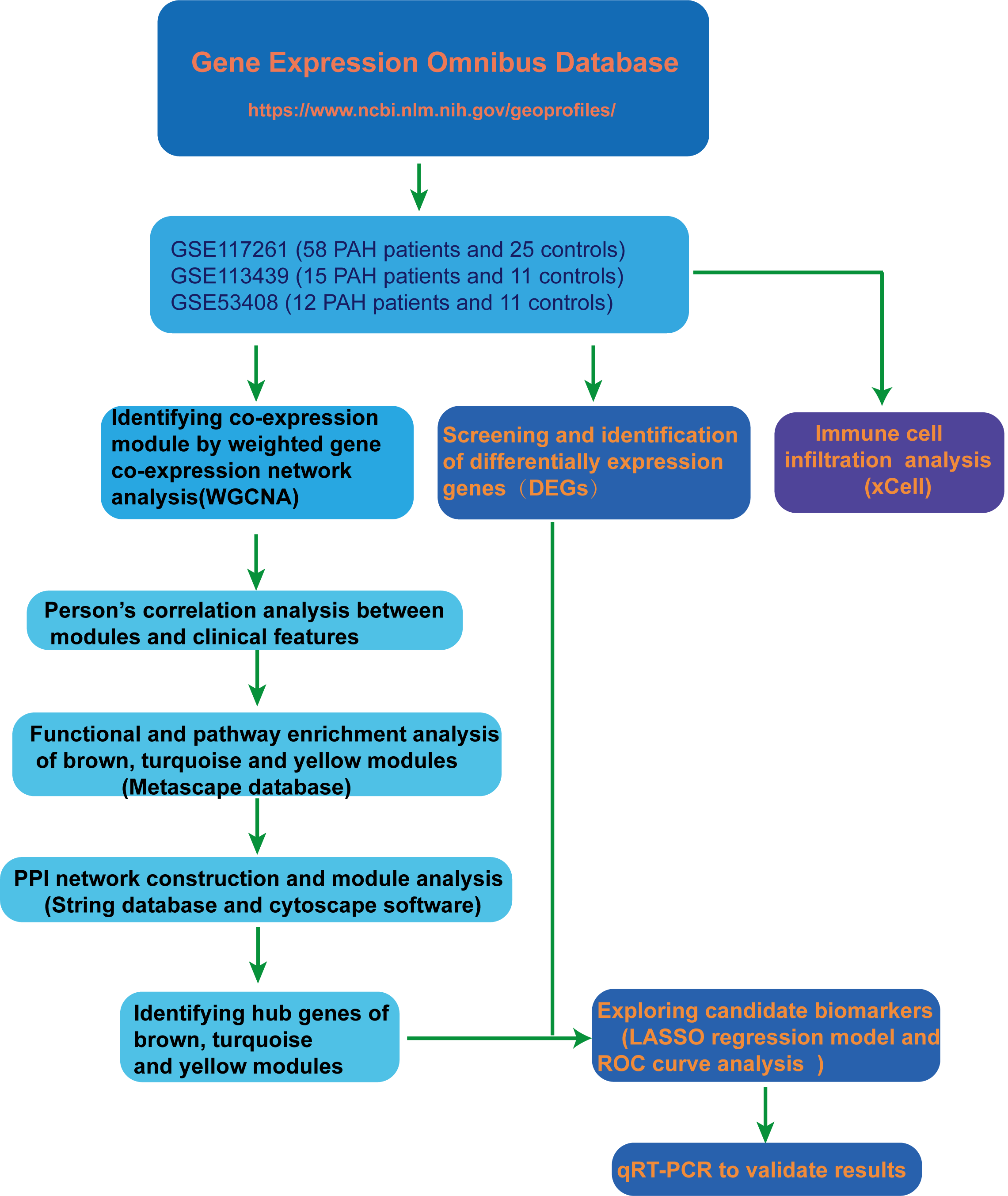


Supplement figure 1

The workflow of this study. PPI, protein-protein interaction.


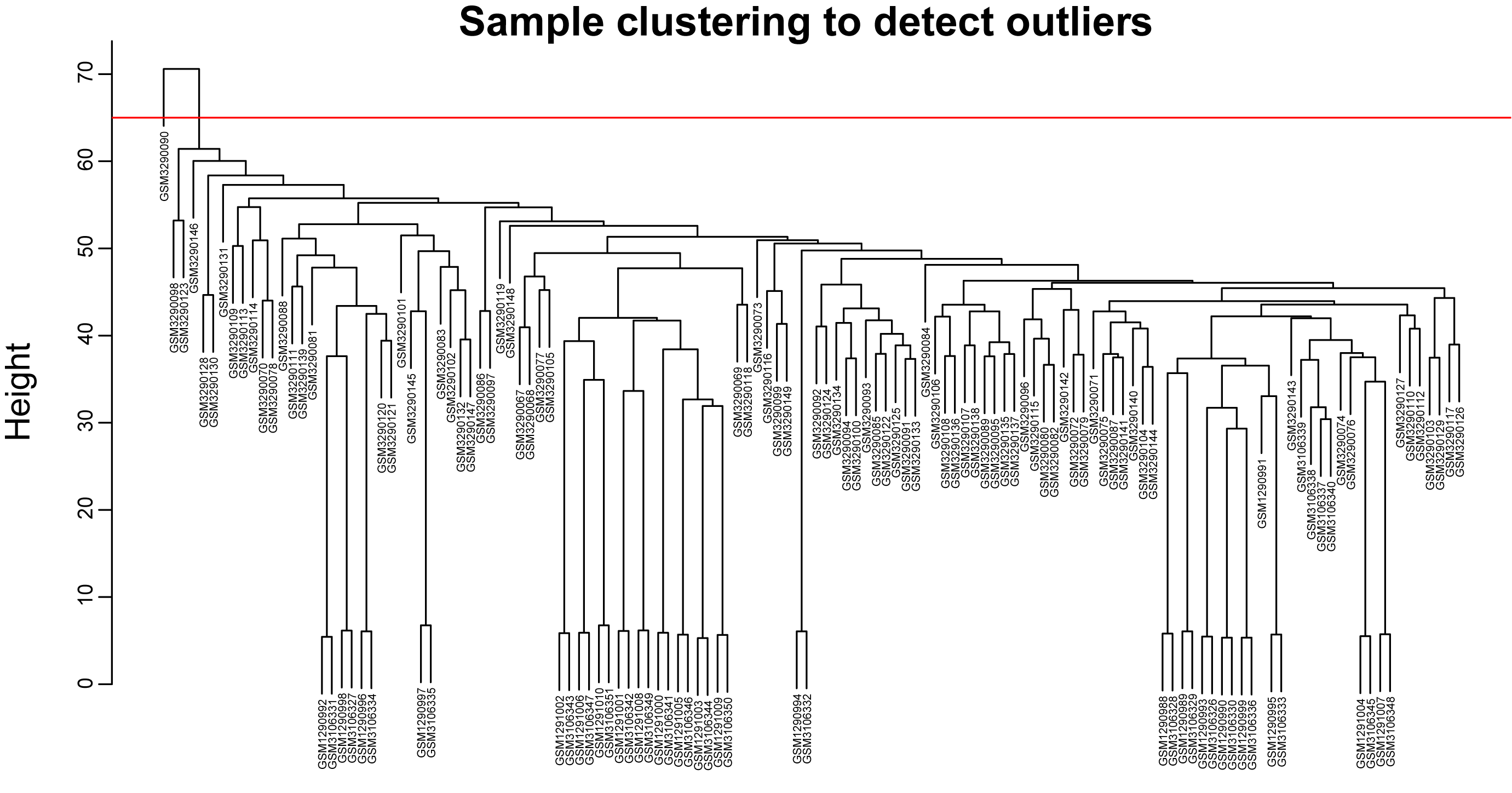


Supplement figure 2

Clustering dendrogram of samples based on Euclidean distance. It shows an

obvious outlier (sample GSM 3290090) that we removed for the following analysis.


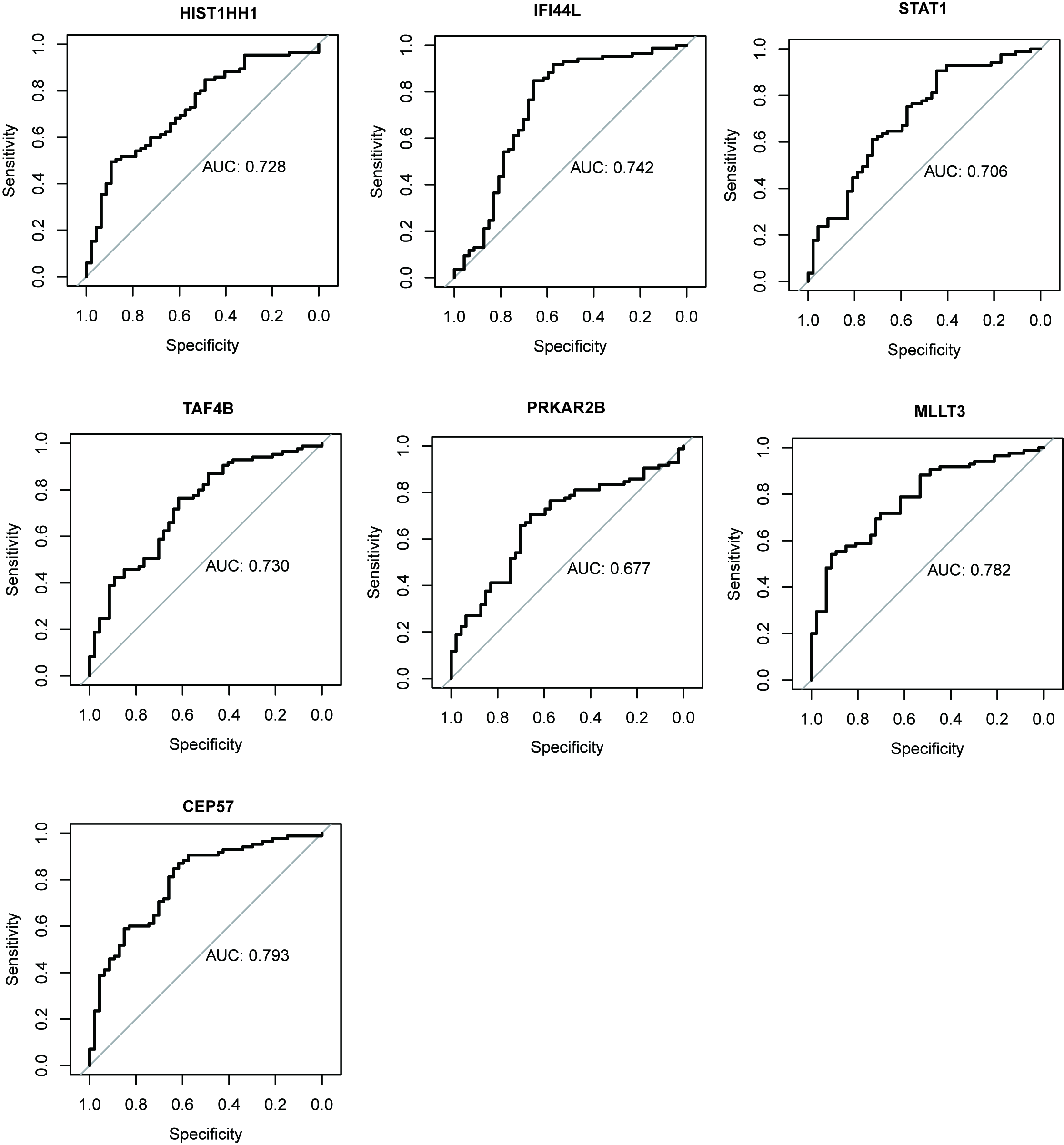


Supplement figure 3

Identification and validation the biomarkers of PAH.

The receiver operating characteristic (ROC) curve of the discrimination ability of *HIST1H1C, IFI44L, STAT1, TAF4B, PRKAR2B, MLLT3*, and *CEP57.* AUC, area under the curve.
